# Development of an early diagnostic model for gastric cancer combining serum cytokine profiles with conventional tumor markers: a case–control study

**DOI:** 10.3389/fonc.2026.1804938

**Published:** 2026-04-28

**Authors:** Qiuyan Zhang, Jing Zhai, Pu Chen, Tiantian Han, Xueting Deng, Lin Miao, Xiuhua Zhang

**Affiliations:** Department of Gastroenterology, Second Affiliated Hospital, Nanjing Medical University, Nanjing, Jiangsu, China

**Keywords:** biomarkers, cytokines, early detection, gastric cancer, precancerous lesions, tumor markers

## Abstract

**Background:**

Early detection of gastric cancer (GC) remains challenging, and conventional serum tumor markers show limited sensitivity for precancerous gastric lesions. Inflammatory cytokines change during tumorigenesis and may provide complementary information for noninvasive screening. We evaluated whether serum cytokine profiling combined with routine tumor markers could improve discrimination across the spectrum from healthy mucosa to precancerous lesions and early GC.

**Methods:**

In this single-center, cross-sectional case–control study, 165 participants were enrolled: 60 patients with GC (including 15 high-grade intraepithelial neoplasia [HGIN]/carcinoma *in situ*), 50 individuals with gastric precancerous lesions (GPL; 25 atrophic gastritis [AG] and 25 intestinal metaplasia [IM]), and 55 healthy controls (HC). Serum levels of nine cytokines (IL-1ra, IL-6, IL-7, IL-8, IL-10, IL-16, IL-17, IL-21, and necrosis factor-α [TNF-α] ) were quantified by ELISA, and five conventional tumor markers (carcinoembryonic antigen [CEA], carbohydrate antigen 19–9 [CA19-9], carbohydrate antigen CA125 [CA125], carbohydrate antigen CA72–4 [CA72-4] andalpha fetoprotein [AFP] ) were recorded. Group comparisons were performed using the Kruskal–Wallis test followed by multiple-testing correction. Diagnostic performance was assessed using receiver operating characteristic (ROC) analysis. Combined models were constructed using multivariable logistic regression and evaluated using predicted probabilities.

**Results:**

Compared with HC, GC cases exhibited higher IL-6, IL-8, IL-10, IL-16, IL-17, IL-21, and TNF-α, and lower IL-1ra and IL-7. GPL cases showed increased IL-16 and IL-17 and decreased IL-7,IL-16 correlated moderately with IL-10(r=0.62), suggesting coordinated inflammatory–immunoregulatory signaling. As individual markers, IL-16 yielded an AUC of 0.91 for GC versus HC, IL-7 yielded an AUC of 0.76 for GC versus GPL, and IL-16 and IL-17 showed value for detecting GPL (AUCs 0.74 and 0.73, respectively). Multimarker combinations improved discrimination: IL-6+IL-7+IL-16+CEA reached an AUC of 0.93 for GC versus GPL; IL-6+IL-16+IL-17 reached an AUC of 0.92 for GPL versus HC; and an integrated model discriminating GC from HC achieved an AUC of 0.97. Bootstrap internal validation showed optimism-corrected AUCs of 0.902, 0.889, and 0.927 for these three combined models, respectively SHAP-based ranking indicated that IL-16, IL-6, CEA, and IL-7 were the main contributors to the 12-analyte model, whereas IL-21 had relatively low marginal contribution.

**Conclusions:**

Serum inflammatory cytokine patterns change early during gastric carcinogenesis. Combining cytokine profiles with conventional tumor markers substantially enhances noninvasive discrimination of precancerous lesions and early GC. However, these findings should be interpreted as exploratory and require multicenter and longitudinal validation before clinical application.

## 1 Introduction

Gastric cancer (GC) remains a major global health burden in both incidence and mortality. According to the 2022 global cancer statistics, newly diagnosed GC accounted for approximately 4.9% of all cancers and 6.8% of all cancer-related deaths worldwide (1). In China, the burden is particularly severe, with both incidence and mortality ranking third among all malignant tumors (2). Early GC often lacks specific clinical manifestations, and approximately 80% of patients are diagnosed at intermediate or advanced stages, resulting in limited treatment options, poor prognosis, and a 5-year survival rate below 50% (2). Therefore, early detection and intervention remain crucial for improving patient outcomes and reducing disease burden.

Gastric carcinogenesis is a prolonged and multifactorial process involving genetic susceptibility, environmental exposures, infection, and lifestyle factors. Helicobacter pylori infection is the most important and preventable risk factor for non-cardia intestinal-type GC (3, 4). Age, male sex, diet, smoking, and alcohol consumption are additional risk factors (3, 5). Histologically, intestinal-type GC often follows the Correa cascade: normal mucosa → chronic gastritis → atrophic gastritis → intestinal metaplasia → intraepithelial neoplasia → invasive carcinoma. Atrophic gastritis and intestinal metaplasia are considered gastric precancerous lesions (GPL) and represent critical windows for early intervention (6).

Although the Correa cascade underlies many prevention strategies, efficient noninvasive approaches for population-level screening of GPLs and early GC remain limited. Upper gastrointestinal endoscopy, the diagnostic gold standard, is constrained in mass screening by its invasiveness, cost, and accessibility. Common serum tumor markers, including carcinoembryonicantigen (CEA), carbohydrate antigen 19-9 (CA19-9), carbohydrate antigen CA125(CA125), and carbohydrate antigen CA72-4(CA72-4), have adjunctive value but insufficient sensitivity and specificity, especially in GPLs and early GC, leading to high false-negative rates (7, 8). Therefore, single tumor markers are inadequate, and more sensitive biomarkers that dynamically reflect disease progression are urgently needed.

The interaction between inflammation and cancer has long been recognized as a central theme in tumor biology (9, 10). The inflammatory tumor microenvironment comprises inflammatory cells, chemokines, cytokines, and signaling pathways, among which inflammatory cytokines act as central mediators of immune regulation and tumor-promoting inflammation (11, 12). For example, IL-6 has been reported to promote gastric cancer growth and progression (13). An Italian study identified IL-8 and tumor necrosis factor-α (TNF-α) as potential diagnostic biomarkers (14). IL-10 exerts context-dependent immunosuppressive effects (15), while IL-16, IL-17, and IL-21 have been implicated in tumor-associated immune regulation (16, 17). Because changes in serum cytokine levels may precede overt morphological alterations, they may have value as early diagnostic biomarkers.

However, most previous studies have focused on single-cytokine mechanisms or on therapeutic targets in advanced disease. Systematic profiling of cytokines across the gastric carcinogenic sequence, particularly at the GPL stage, remains limited. Integrative studies combining cytokine profiles with conventional tumor markers to improve early discrimination are still scarce, which hinders clinical translation.

Accordingly, we hypothesized that combining serum cytokines with routine tumor markers could improve discrimination of GPLs and early GC. To test this hypothesis, we measured serum levels of nine cytokines (IL-1ra, IL-6, IL-7, IL-8, IL-10, IL-16, IL-17, IL-21, and TNF-α) and five conventional tumor markers (CEA, CA125, CA19-9, CA72-4, and alpha fetoprotein (AFP) in patients with GC, patients with GPL, and healthy controls. Through intergroup comparisons, exploratory correlation analyses, and receiver operating characteristic ( ROC ) -based diagnostic modeling, we aimed to identify marker combinations with potential value for early screening and risk stratification.

## 2 Materials and methods

### 2.1 Study design and participants

This study was designed as a single-center, cross-sectional case–control investigation, intended primarily for exploratory biomarker discovery and disease-stage discrimination rather than temporal or causal inference. Between June 2024 and June 2025, a total of 165 consecutive participants who underwent diagnostic esophago-gastroduodenoscopy and had histopathological confirmation at the Second Affiliated Hospital of Nanjing Medical University were enrolled, including 60 patients with GC, 50 patients with GPL, and 55 healthy controls (HC).

Within the GPL group, 25 patients had atrophic gastritis (AG) and 25 had intestinal metaplasia (IM). Within the GC group, 15 patients had high-grade intraepithelial neoplasia (HGIN; carcinoma *in situ*), and 45 had invasive gastric cancer. HGIN/carcinoma *in situ* was included in the main GC group for the primary analysis. In addition, we performed an exploratory subgroup comparison between HGIN and invasive GC to assess whether significant serum biomarker differences existed between the earliest malignant stage and invasive disease (**Supplementary Table S9**). The study protocol was approved by the Ethics Committee of the Second Affiliated Hospital of Nanjing Medical University (approval no. 2025-KY-183-01) and was conducted in accordance with the principles of the Declaration of Helsinki. Written informed consent was obtained from all participants; for individuals lacking full decision-making capacity, consent was obtained from their legally authorized representatives.

The inclusion criteria for the GC group were as follows: (1) age > 18 years; (2) diagnosis of gastric cancer confirmed by endoscopy and histopathology; and (3) no prior radiotherapy, chemotherapy, immunotherapy, or other antitumor treatment before enrollment. The inclusion criteria for the GPL group were: (1) age > 18 years; and (2) histopathological confirmation of atrophic gastritis and/or intestinal metaplasia. Exclusion criteria included: (1) concurrent other malignancies; (2) prior antitumor treatment; (3) a history of gastric surgery or endoscopic resection; (4) coexisting acute or chronic inflammatory diseases unrelated to the gastric lesion; (5) severe dysfunction of major organs; and (6) severe cognitive impairment affecting compliance.

### 2.2 Collection of baseline data

Baseline data for all participants were collected from medical records and a standardized questionnaire, including age, sex, body mass index (BMI), smoking history, alcohol consumption history, Helicobacter pylori infection status, metabolism-related diseases, and family history of gastrointestinal malignancy.BMI was categorized as < 18.5 kg/m², 18.5–23.9 kg/m², 24.0–27.9 kg/m², and ≥28.0 kg/m². It should be noted that BMI categories were used for descriptive baseline comparison only and were not intended to replace continuous covariate adjustment in statistical models.Smoking history was defined as cumulative smoking of at least 100 cigarettes and a duration of at least 6 months. Alcohol consumption history was defined as drinking at least three times per week for at least 1 year. Metabolism-related diseases included clinically diagnosed diabetes mellitus, dyslipidemia, and hyperuricemia. A family history of gastrointestinal malignancy referred to a history of relevant malignancy in first-degree relatives.H. pylori infection status was determined based on histopathology, serological detection of urease antibodies, and the ^13C/^14C urea breath test. A delta-over-baseline (DOB) value ≥4.0 in the breath test was considered positive.

### 2.3 Blood collection and laboratory assessment

A 5 mL fasting peripheral venous blood sample was collected from each participant in the morning. Blood samples were allowed to clot at room temperature and were then centrifuged at 900 × g for 8 min to separate serum. The serum was aliquoted into sterile low-temperature storage tubes and stored at −80 °C until analysis.Serum levels of IL-1ra, IL-6, IL-7, IL-8, IL-10, IL-16, IL-17, IL-21, and TNF-α were measured using commercial ELISA kits (Jianglai Biotechnology Co., Ltd., China) according to the manufacturer’s instructions.

To ensure analytical reliability, all serum samples were aliquoted after collection, stored at −80 °C, and analyzed together under uniform experimental conditions in a single analytical phase. All nine cytokines were measured in duplicate, and the mean of the two measurements was used for subsequent statistical analysis. Samples with variation between duplicate wells exceeding the predefined acceptable threshold were re-assayed. In addition, spike-in recovery experiments were performed in representative serum samples by adding known concentrations of cytokine standards to evaluate assay accuracy and potential matrix effects. Detailed quality-control metrics are provided in **Supplementary Table S1**.

Data for conventional tumor markers, including CEA, CA125, CA19-9, CA72-4, and AFP, were extracted from the same institutional clinical laboratory system. Because all participants were enrolled at a single center and all corresponding assays were performed within the same institutional laboratory system, cross-laboratory technical heterogeneity was minimized by study design.

### 2.4 Statistical analysis

Because most biomarker data were not normally distributed, nonparametric statistical methods were used. Overall group differences were assessed using the Kruskal–Wallis H test. When the overall test was significant, Dunn’s *post hoc* test was applied for pairwise comparisons. Multiple comparisons were controlled using the Benjamini–Hochberg false discovery rate (FDR) procedure, and adjusted q-values were reported where appropriate. To complement inference based solely on P values, nonparametric effect sizes were additionally calculated. Specifically, epsilon-squared (ϵ²) was used as the overall effect-size estimate for Kruskal–Wallis tests, and Cliff’s delta was used as the effect-size measure for pairwise comparisons. The corresponding results are presented in **Supplementary Tables S3**, **S4**.

Associations between cytokines and tumor markers were evaluated using Spearman rank correlation analysis. It should be emphasized that this correlation analysis was exploratory in nature. For major correlation pairs, FDR correction was additionally performed, and the corresponding results are shown in **Table 1**.

**Table 1:** Spearman correlations between serum cytokines and tumor markers in the gastric cancer group.

Biomarker1	Biomarker2	Spearman r	Raw P-value	FDR-adjusted q-value	Strength of correlation
IL-16	IL-10	0.62	0.005	0.023	Significant
IL-8	CA724	0.57	0.004	0.022	Significant
CEA	CA724	0.55	0.009	0.031	Significant
IL-16	IL-1ra	0.43	0.018	0.052	Trend
IL-16	CEA	0.52	0.019	0.052	Trend
TNF-α	IL-21	0.50	0.026	0.061	Trend
CA199	IL-8	0.51	0.027	0.061	Trend
IL-6	IL-21	0.46	0.042	0.084	NS
TNF-α	IL-17	-0.41	0.036	0.079	NS
IL-16	CA724	0.38	0.032	0.074	NS

Only correlation pairs with raw P < 0.05 are shown in the main table. Significant: q < 0.05; Trend: 0.05 ≤ q < 0.10; NS: q ≥ 0.10.

ROC analysis was used to assess the discriminatory performance of individual biomarkers and combined biomarker models. The area under the curve (AUC) was used to quantify diagnostic accuracy. No formal *a priori* sample size calculation based on AUC was performed at the study design stage. However, for biomarkers with AUC ≥ 0.70, we conducted a *post-hoc* power assessment for the three prespecified pairwise comparisons (GC vs HC, GPL vs HC, and GC vs GPL), and the results are presented in **Supplementary Table S2**. For each pairwise ROC comparison, we additionally calculated the Youden index (J), the corresponding optimal cutoff, and the sensitivity at a fixed specificity of 90% to improve the clinical interpretability of the ROC results.

For multivariable combined biomarker models, logistic regression was used to construct the models, and ROC analysis was performed based on the predicted probabilities. To evaluate model robustness, bootstrap internal validation with 1,000 resamples was performed. Apparent AUC, average optimism, optimism-corrected AUC, and percentile-based 95% confidence intervals were reported (**Supplementary Table S7**). For the 12-analyte combined model, SHAP (Shapley additive explanations) analysis was additionally performed to evaluate variable importance and improve model interpretability (**Supplementary Table S8**).Unless otherwise specified, a two-sided P value < 0.05 was considered statistically significant.

## 3 Result

### 3.1 Baseline characteristics of participants

This study enrolled 165 participants: 60 patients with GC, 50 with GPL—25 with atrophic gastritis (AG) and 25 with intestinal metaplasia (IM)—and 55 healthy controls (HC). As shown in **Table 2**, there were no significant differences among the groups in age (P = 0.062), sex (P = 0.510), body mass index (BMI) category (P = 0.379), smoking (P = 0.230), alcohol consumption (P = 0. 191), Helicobacter pylori infection status (P = 0.780), prevalence of metabolic comorbidities (P = 0.649), or family history of gastrointestinal malignancies (P = 0.210). These findings indicate balanced baseline characteristics and minimize potential confounding in subsequent serum biomarker analyses.H. pylori positivity was similarly distributed across the three groups (approximately 50% in each group), suggesting no major between-group imbalance in infection status at baseline.

**Table 2:** Summary of clinical characteristics of study groups.

Parameter	GC(n=60)	GPL(n=50)		HC(n=55)	P
		AG (n=25)	IM (n=25)		
Age ,years Sex, N (%)	62.1 ± 8.0	59.6 ± 4.2	56.2 ± 6.4	55.6 ± 5.6	0.062
Male	23 (38.3)	12 (48.0)	13 (52.0)	28 (50.9)	
Female	37 (61.7)	13 (52.0)	12 (48.0)	27 (49. 1)	0.510
BMI, N (%)					
< 18.5 kg/m 2	12 (20.0)	5 (20.0)	4 (16.0)	9 (16.4)	
18.5–23.9 kg/m 2	28 (46.7)	12 (48.0)	11 (44.0)	32 (58.2)	
24-27.9 kg/m 2	18 (30.0)	7 (28.0)	9 (36.0)	13 (23.6)	
≥ 28 kg/m 2	2 (3.3)	1 (4.0)	1 (4.0)	1(1.8)	0.379
Ever smoking, N (%)					
Yes	23 (38.3)	9 (36.0)	9 (36.0)	13 (23.6)	
No	37 (61.7)	16 (64.0)	16 (64.0)	42 (76.4)	0.230
Ever drinking, N (%)					
Yes	19 (31.7)	10 (40.0)	9 (36.0)	12 (21.8)	
No	41 (68.3)	15 (60.0)	16 (64.0)	43 (78.2)	0.191
H.pylori infection, N (%)					
Positive	30 ( 50.0)	14 ( 56.0)	11 (44.0)	25 (0.45)	
Negative	30 ( 50.0)	11 (44.0)	14 (56.0)	30 (55.0)	0.780
Metabolism-related diseases, N (%)					
Yes	15 (25.0)	7 (28.0)	5 (20.0)	10 (18. 1)	
No	45 (66.7)	18 ( 72.0)	20 (80.0)	45 (81.8)	0.649
Family history of gastrointestinal cancer, N (%)					
Yes	13 (21.7)	3 (12.0)	2 (8.0)	6 (10.9)	
No	47 ( 78.3)	22 (88.0)	23 (92.0)	49 (89. 1)	0.210

Continuous variables are presented as mean ± standard deviation. Categorical variables are presented as number (percentage). BMI categories were used for descriptive baseline comparison only and were not intended to replace continuous covariate adjustment in analytical models. Metabolism-related diseases include clinically diagnosed diabetes mellitus, dyslipidemia, and hyperuricemia. P values were calculated for overall between-group comparisons.

Continuous variables are presented as mean ± standard deviation. Categorical variables are presented as number (percentage). BMI categories were used for descriptive baseline comparison only and were not intended to replace continuous covariate adjustment in analytical models. Metabolism-related diseases include clinically diagnosed diabetes mellitus, dyslipidemia, and hyperuricemia. P values were calculated for overall between-group comparisons.

### 3.2 Serum cytokine and tumor marker differences

**Figure 1:**
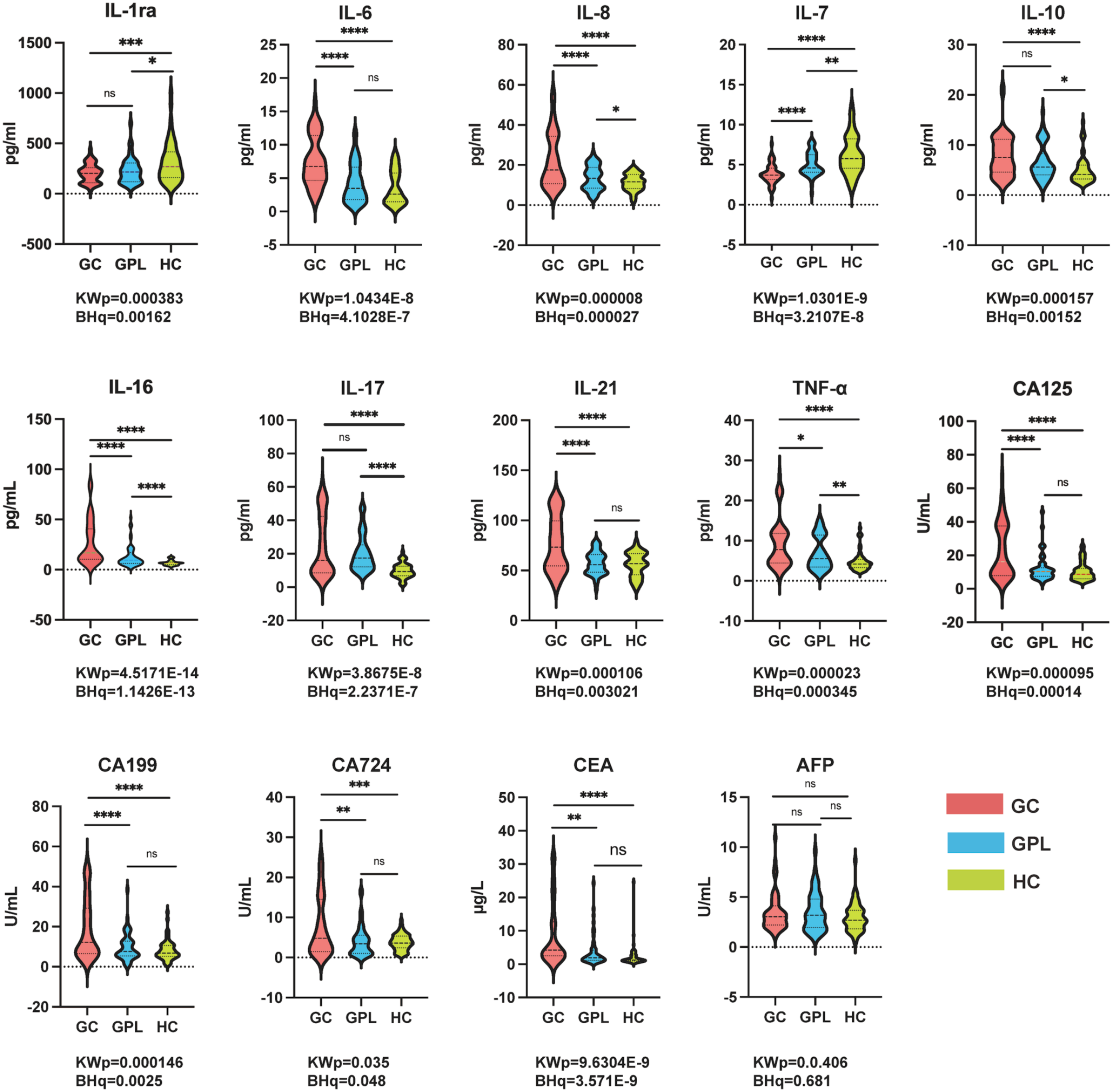
Comparison of plasma analyte concentrations was performed among patients with gastric cancer, patients with precancerous gastric lesions, and healthy controls. Kruskal–Wallis (KW) p-values and Benjamini–Hochberg (BH)–adjusted q-values were used for overall between-group comparisons; when the Kruskal–Wallis p-value was significant, Bonferroni-corrected p-values were applied for pairwise comparisons. Significance is indicated as: ns, p ≥ 0.05; *p < 0.05; **p < 0.01; ***p < 0.001; ****p < 0.0001. BH, Benjamini–Hochberg; TNF-α, tumor necrosis factor alpha; CA125, cancer antigen 125; CA199, carbohydrate antigen 199; CA724, carbohydrate antigen 724; CEA, carcinoembryonic antigen; AFP, alpha-fetoprotein.

Serum concentrations of nine cytokines and five tumor markers were compared among GC, GPL, and HC groups using the Kruskal–Wallis H test. As shown in **Table 3**, **Figure 1**, most analytes differed significantly among groups after multiple-testing correction. Additional overall nonparametric effect-size estimates are provided in **Supplementary Table S3**.

**Table 3:** Overall between-group comparison results for serum cytokines and tumor markers using the Kruskal–Wallis test.

Biomarker	Kruskal–Wallis H (K)	Asymptotic significance	Adjusted significance
CA125	18.521	0.000095	0.00014
CA199	17.663	0.000146	0.00253
CA724	6.683	0.035	0.048
CEA	36.917	9.6304E-9	3.571E-9
AFP	1.801	0.406	0.681
IL-16	61.457	4.5171E-14	1.1426E-13
IL-6	36.756	1.0434E-8	4.1028E-7
IL-8	23.428	0.000008	0.000027
IL-10	17.517	0.000157	0.00152
IL-17	34.136	3.8675E-8	2.2371E-7
TNF-α	21.400	0.000023	0.000345
IL-7	41.387	1.0301E-9	3.2107E-8
IL-21	18.302	0.000106	0.003021
IL-1ra	15.737	0.000383	0.00162

Compared with HC, GC patients had higher serum levels of IL-6, IL-8, IL-10, IL-16, IL-17, IL-21, and TNF-α (all P<0.0001), whereas IL-1ra and IL-7 were lower (both P<0.001). Relative to the GPL group, GC patients showed elevated IL-6, IL-8, IL-16, IL-21 (all P<0.0001) and increased TNF-α (P<0.05), with reduced IL-7 (P<0.0001). IL-1ra, IL-10, and IL-17 did not differ significantly between GC and GPL.GPL patients exhibited higher IL-16 and IL-17 versus HC (both P<0.0001), with modest increases in IL-8, IL-10, and TNF-α (P<0.05). IL-1ra and IL-7 were lower in GPL than in HC, while IL-6 and IL-21 did not differ significantly (**Figure 1**). Overall, cytokine profiles showed progressive alterations from HC to GPL to GC, with IL-6, IL-8, IL-16, and IL-21 most markedly elevated in GC and IL-7 declining across stages.

### 3.3 Tumor marker profiles

GC patients had higher serum CA125, CA199, CA724, and CEA than HC (all P<0.001); AFP did not differ significantly among groups. Compared with GPL, GC patients also showed increased CA125 and CA199 (both P<0.0001) and elevated CA724 and CEA (both P<0.01). Tumor marker levels did not differ significantly between GPL and HC. These results indicate that conventional tumor markers better distinguish GC from healthy individuals but have limited sensitivity for detecting precancerous lesions.

This table summarizes overall between-group comparison results rather than descriptive statistics for each individual group. “Asymptotic significance” indicates the overall Kruskal–Wallis P value, and “Adjusted significance” indicates the multiple-testing–adjusted P value.

### 3.4 Overall effect sizes and pairwise IL-10 effect patterns

Overall nonparametric effect-size analysis using epsilon-squared showed that IL-16 exhibited the largest group effect (ϵ² = 0.367), followed by IL-7 (ϵ² = 0.244), CEA (ϵ² = 0.216), IL-6 (ϵ² = 0.214), and IL-17 (ϵ² = 0. 198). In contrast, IL-10 showed a moderate overall effect size (ϵ² =0.096), suggesting that its group differences were more subtle and may be less consistently detectable in pairwise comparisons (**Supplementary Table S3**).

For IL-10, pairwise Cliff’s delta analysis showed a medium effect for GC vs HC (δ = 0.383), a large effect for GPL vs HC (δ = 0.596), and only a small effect for GC vs GPL (δ = −0.242) (**Supplementary Table S4**). These findings indicate that IL-10 differences were more pronounced between disease groups and healthy controls than between GC and GPL, which may explain why some pairwise comparisons were less stable or more difficult to detect statistically.

### 3.5 Exploratory subgroup analysis within GPL: AG versus IM

To further explore heterogeneity within the GPL group, we performed an exploratory subgroup comparison between AG and IM. No statistically significant differences were observed between AG and IM for any measured cytokine after multiple-testing correction, including IL-17 (**Supplementary Table S5**). Likewise, conventional tumor markers did not differ significantly between the two subgroups (**Supplementary Table S6**). These findings suggest that AG and IM were not detectably separated by the current biomarker panel at the present sample size.

### 3.6 Exploratory subgroup analysis within GC: HGIN/Carcinoma *in situ* versus invasive GC

To assess whether pooling HGIN/carcinoma *in situ* with invasive GC may have obscured stage-specific serum differences, we performed an exploratory subgroup analysis comparing HGIN/carcinoma *in situ* with invasive GC across all measured serum biomarkers. No statistically significant differences were identified for any cytokine or tumor marker after multiple-comparison correction (**Supplementary Table S9**).

### 3.7 Correlation between cytokines and tumor markers

**Figure 2:**
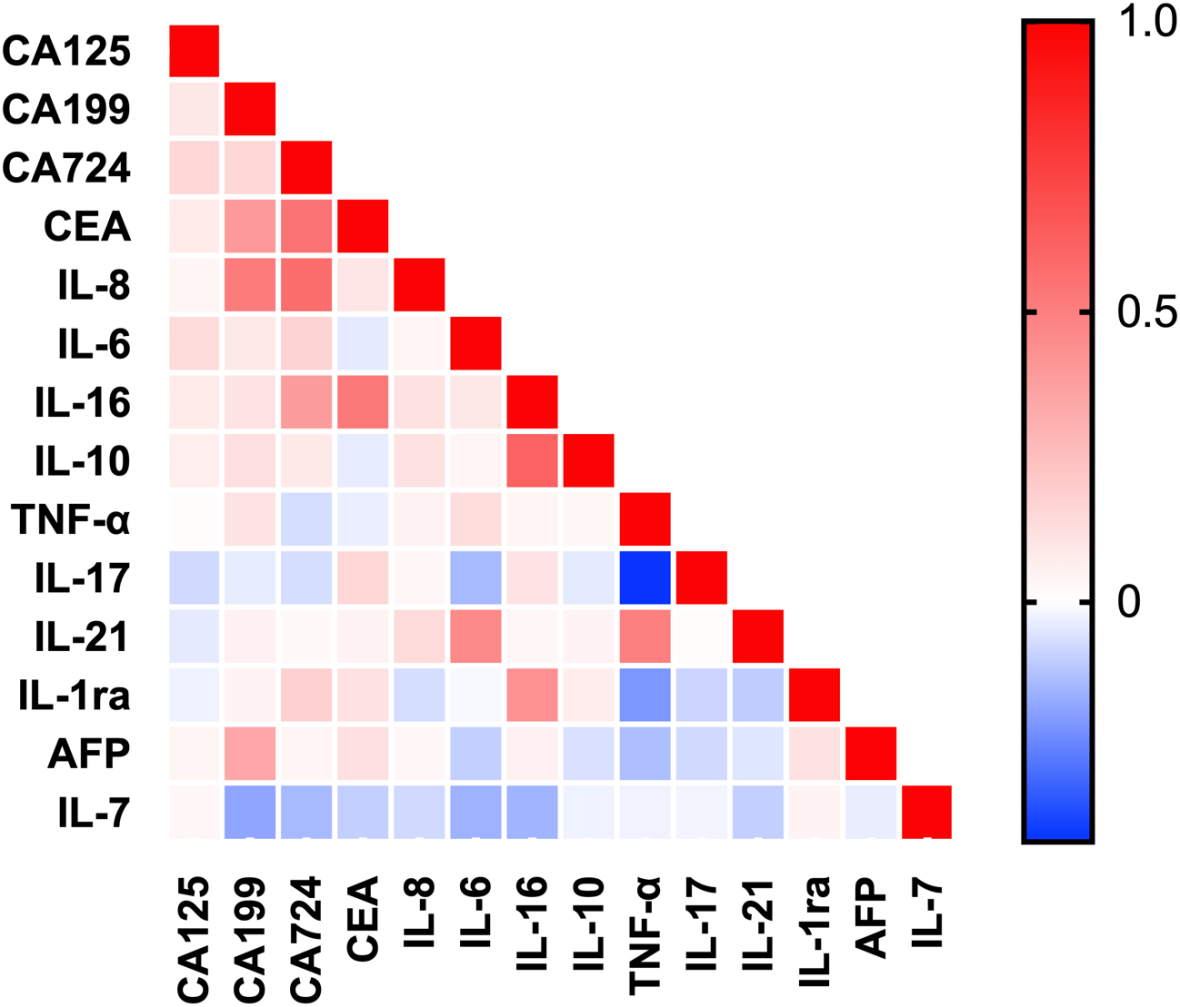
Heatmap of correlation levels between cytokines and tumor markers. Spearman correlation coefficients were used; *indicates P < 0.05 (trend) and **indicates P < 0.01 (significant).

Spearman correlation analysis in GC patients identified multiple significant associations between cytokines and tumor markers (**Figure 2**, **Table 1**). IL-16 correlated strongly with IL-10 (r= 0.62, P = 0.005). Moderate positive correlations (r = 0.43–0.57) were observed for IL-8 with CA724, CEA with CA724, IL-16 with CEA, CA199 with IL-8, TNF-α with IL-21, IL-6 with IL-21, and IL-16 with IL-1ra. TNF-α was moderately negatively correlated with IL-17 (r=−0.41, P = 0.036). Additional weaker positive correlations suggest a complex, sometimes opposing, coordination of cytokine and tumor marker expression in GC.

However, these analyses were exploratory and unadjusted and should not be interpreted as fully adjusted conditional relationships.After FDR correction, only a limited number of pairwise correlations remained statistically significant, including IL-16:IL-10, IL-8:CA72-4, and CEA : CA72-4.

### 3.8 Diagnostic performance of serum biomarkers

**Figure 3:**
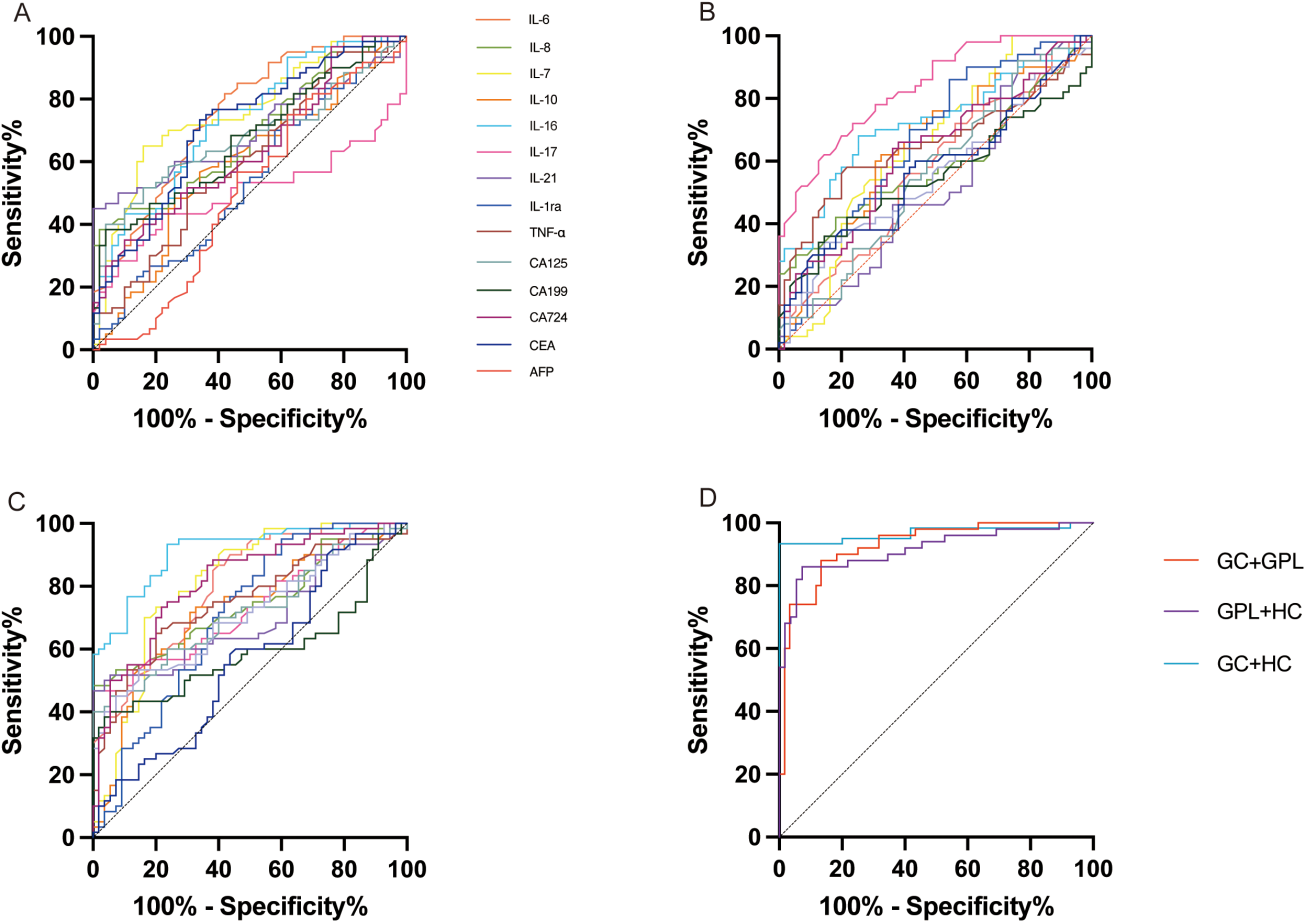
Receiver operating characteristic (ROC) curves of plasma analytes were used to discriminate among subject groups. **(A)** patients with gastric cancer versus patients with precancerous gastric lesions; **(B)** patients with precancerous gastric lesions versus healthy controls; **(C)** patients with gastric cancer versus healthy controls; **(D)** combined biomarkers with area under the ROC curve (AUC) > 0.7 identified in **(A–C)**.

ROC analysis assessed the diagnostic accuracy of individual biomarkers for discriminating GC, GPL, and HC (**Tables 4**-**6**, **Figures 3A–C**). In distinguishing GC from GPL (**Table 4**,**Figure 3A**), IL-7 (AUC = 0.76), IL-16 (AUC = 0.75), IL-6 (AUC = 0.74), and CEA (AUC = 0.71) had the highest accuracy. IL-8, IL-21, CA125, and CA19–9 also demonstrated significant discrimination, whereas IL-10, IL-17, IL-1ra, TNF-α, and AFP performed poorly.For GPL versus HC (**Table 5**, **Figure 3B**), cytokines outperformed tumor markers: IL-16 (AUC = 0.74), IL-17 (AUC = 0.73), and IL-6 (AUC = 0.72) showed diagnostic potential, while no tumor marker exceeded an AUC of 0.63.In distinguishing GC from HC (**Table 6**, **Figure 3C**), IL-16 exhibited the highest accuracy (AUC = 0.91), followed by IL-17, CEA, IL-7, and IL-6 (AUCs =0.80–0.83). CA724 and AFP showed limited and non-significant discrimination.

**Table 4:** Diagnostic performance of individual biomarkers for distinguishing gastric cancer from precancerous lesions (GC vs GPL).

Biomarker	Comparison	AUC (95% CI)	P	Youden index (J)	Optimal cutoff	Sensitivity at 90% specificity
IL-6	GC vs GPL	0.74 (0.66-0.84)	<0.0001	0.44	> 11.23 pg/mL	0.52
IL-8	GC vs GPL	0.69 (0.59-0.78)	0.0008	0.34	> 20.17 pg/mL	0.23
IL-7	GC vs GPL	0.76 (0.67-0.85)	<0.0001	0.46	≤ 3.85 pg/mL	0.58
IL-10	GC vs GPL	0.59 (0.48-0.67)	0.1031	0.20	> 14.56pg/mL	0.13
IL-16	GC vs GPL	0.75 (0.64-0.82)	<0.0001	0.45	> 14.75 pg/mL	0.43
IL-17	GC vs GPL	0.50 (0.39-0.61)	0.992	0.09	> 45.67 pg/mL	0.05
IL-21	GC vs GPL	0.69 (0.60-0.80)	0.004	0.34	> 115.98 pg/mL	0.28
IL-1ra	GC vs GPL	0.54 (0.43-0.65)	0.446	0.11	> 341.78 pg/mL	0.08
TNF- α	GC vs GPL	0.60 (0.50-0.71)	0.064	0.22	> 31.45 pg/mL	0.10
CA125	GC vs GPL	0.67 (0.57-0.77)	0.0018	0.30	> 36.88 u/mL	0.25
CA199	GC vs GPL	0.67 (0.57-0.77)	0.002	0.32	> 27.50 u/mL	0.28
CA724	GC vs GPL	0.64 (0.54-0.74)	0.0132	0.24	> 7.02 u/mL	0.18
CEA	GC vs GPL	0.71 (0.62-0.81)	0.0001	0.40	> 3.41 ng/mL	0.33
AFP	GC vs GPL	0.50 (0.39-0.61)	0.9928	0.08	> 3.57 ng/mL	0.07

AUC, area under the curve; CI, confidence interval. The Youden index (J), optimal cutoff, and sensitivity at 90% specificity are shown to improve the clinical interpretability of ROC performance.

**Table 5:** Diagnostic performance of individual biomarkers for distinguishing precancerous lesions from healthy controls (GPL vs HC).

Biomarker	Comparison	AUC (95% CI)	P	Youden index (J)	Optimal cutoff	Sensitivity at 90% specificity
IL-6	GPL vs HC	0.72 (0.66-0.84)	<0.0001	0.41	> 2.84 pg/mL	0.28
IL-8	GPL vs HC	0.59 (0.48-0.70)	0.1116	0.18	> 15.33 pg/mL	0.12
IL-7	GPL vs HC	0.65 (0.54-0.75)	0.0096	0.28	≤ 5.64 pg/mL	0.24
IL-10	GPL vs HC	0.65 (0.54-0.75)	0.0104	0.24	> 6.49 pg/mL	0.14
IL-16	GPL vs HC	0.74 (0.62-0.82)	0.0001	0.44	> 7.89 pg/mL	0.42
IL-17	GPL vs HC	0.73 (0.64-0.82)	<0.0001	0.43	> 11.45 pg/mL	0.32
IL-21	GPL vs HC	0.52 (0.41-0.63)	0.7362	0.06	> 57.90pg/mL	0.02
IL-1ra	GPL vs HC	0.67 (0.56-0.77)	0.0035	0.30	> 216.40 pg/mL	0.20
TNF- α	GPL vs HC	0.65 (0.54-0.76)	0.0078	0.26	> 12.15 pg/mL	0.18
CA125	GPL vs HC	0.56 (0.45-0.70)	0.2956	0.14	> 15.60u/mL	0.10
CA199	GPL vs HC	0.56 (0.44-0.67)	0.3301	0.12	> 11.45u/mL	0.08
CA724	GPL vs HC	0.55 (0.43-0.66)	0.3846	0.11	> 5.20 u/mL	0.08
CEA	GPL vs HC	0.63 (0.52-0.73)	0.264	0.24	> 2.47 ng/mL	0.16
AFP	GPL vs HC	0.57 (0.46-0.68)	0.2315	0.15	> 3.40 ng/mL	0.11

**Table 6:** Diagnostic performance of individual biomarkers for distinguishing gastric cancer from healthy controls (GC vs HC).

Biomarker	Comparison	AUC (95% CI)	P	Youden index (J)	Optimal cutoff	Sensitivity at 90% specificity
IL-6	GC vs HC	0.80 (0.72-0.88)	<0.0001	0.55	> 6.51 pg/mL	0.52
IL-8	GC vs HC	0.74 (0.65-0.83)	<0.0001	0.45	> 16.78 pg/mL	0.40
IL-7	GC vs HC	0.81 (0.73-0.89)	<0.0001	0.56	≤ 4.56 pg/mL	0.60
IL-10	GC vs HC	0.72 (0.63-0.82)	<0.0001	0.40	> 12.01 pg/mL	0.30
IL-16	GC vs HC	0.91 (0.86-0.96)	<0.0001	0.79	> 9.87 pg/mL	0.88
IL-17	GC vs HC	0.83 (0.76-0.91)	<0.0001	0.59	> 15.40 pg/mL	0.55
IL-21	GC vs HC	0.70 (0.64-0.82)	0.0004	0.40	> 99.50 pg/mL	0.35
IL-1ra	GC vs HC	0.72 (0.61-0.80)	0.0002	0.38	> 279.57 pg/mL	0.32
TNF- α	GC vs HC	0.75 (0.66-0.84)	<0.0001	0.48	> 21.09 pg/mL	0.42
CA125	GC vs HC	0.72 (0.62-0.81)	<0.0001	0.38	> 27.50 u/mL	0.33
CA199	GC vs HC	0.71 (0.60-0.82)	<0.0001	0.36	> 25.70 u/mL	0.30
CA724	GC vs HC	0.59 (0.49-0.70)	0.0836	0.19	> 7.02 u/mL	0.15
CEA	GC vs HC	0.82 (0.74-0.89)	<0.0001	0.58	> 2.91 ng/mL	0.55
AFP	GC vs HC	0.56 (0.46-0.67)	0.2592	0.11	> 3.57 ng/mL	0.09

For each pairwise ROC comparison, the Youden index (J), the corresponding optimal cutoff, and the sensitivity at 90% specificity were additionally calculated. These analyses showed that optimal cutoffs were comparison-specific rather than universal. For example, the optimal cutoff for IL-16 differed across GC vs GPL, GPL vs HC, and GC vs HC, and IL-7 likewise showed distinct cutoffs across these three comparison settings. In many cases, sensitivity at 90% specificity was lower than the unconstrained optimum implied by the Youden index, highlighting the tradeoff between false-positive control and case detection.

### 3.9 Diagnostic performance of combined biomarker models

Stage-specific multivariate models were constructed using biomarkers with AUC >0.70 (**Table 7**, **Figure 3D**). A model combining IL-6, IL-7, IL-16, and CEA discriminated GC from GPL with excellent performance (AUC = 0.93). A cytokine-based model of IL-6, IL-16, and IL-17 differentiated GPL from HC effectively (AUC = 0.92). An integrated model incorporating all high-performing biomarkers achieved near-perfect accuracy for distinguishing GC from HC (AUC = 0.97). These results suggest that combined quantification of cytokines and tumor markers markedly improves detection across stages of gastric carcinogenesis.Bootstrap internal validation using 1,000 resamples showed that all combined models retained good discriminative performance after optimism correction (**Supplementary Table S7**). To improve interpretability of the 12-analyte model, SHAP-based variable-importance analysis was performed. IL-16, IL-6, CEA, and IL-7 were identified as the dominant contributors to model output, whereas IL-21 had relatively low marginal contribution, and AFP contributed minimally (**Supplementary Table S8**).

**Table 7:** Diagnostic performance of combined biomarker panels.

Biomarker	Comparison	AUC (95% CI)	P	Youden index (J)	Optimal cutoff	Sensitivity at 90% specificity
IL-6+IL-7+IL-16+C EA	GC vs GPL	0.93(0.88-0.98)	<0.0001	0.81	>0.51	0.82
IL-6+IL-16+IL-17	GPL vs HC	0.92(0.86-0.97)	<0.0001	0.78	>0.43	0.71
IL-6+IL-8+IL-7+IL- 10+IL-16+IL-17+IL- 21+IL-1ra+TNF-α+C A 125+CA199+CEA	GC vs HC	0.97(0.93-1.00)	<0.0001	0.93	>0.62	0.98

Combined models were generated using multivariable logistic regression based on selected biomarkers. Bootstrap internal validation results are provided in **Supplementary Table S7**.

## 4 Discussion

We measured serum inflammatory cytokines and conventional tumor markers in patients with gastric cancer (GC), individuals with gastric precancerous lesions (GPL), and healthy controls (HC). The results revealed dynamic changes in the immune microenvironment during gastric mucosal carcinogenesis, and our preliminary evaluation suggests that multimarker combinations may contribute to early detection and risk stratification.

Proinflammatory and immunoregulatory cytokines exhibited stage-specific dynamic changes during gastric carcinogenesis. Serum levels of IL-6, IL-8, IL-10, IL-16, IL-17, IL-21, and TNF-α were significantly elevated in patients with GC. Among these, IL-6, IL-8, IL-16, and IL-21 also differed significantly between the GC and GPL groups, suggesting potential involvement in the transition from precancerous mucosa to malignant disease. As a proinflammatory cytokine, IL-6 promotes tumor cell proliferation, angiogenesis, and immune escape through STAT3 activation (18). Multiple studies have linked elevated IL-6 levels to gastric cancer risk (19, 20). Notably, Helicobacter pylori infection can increase systemic IL-6 levels, and such elevation has been positively associated with GC risk (21). H. pylori may induce IL-6-mediated autocrine and paracrine positive-feedback loops between macrophages and gastric epithelial cells, thereby contributing to gastric carcinogenesis (22). IL-8, a CXC chemokine, reshapes the tumor microenvironment by recruiting neutrophils and promoting angiogenesis (23, 24). Compared with matched normal tissues, gastric cancer tissues show marked upregulation of IL-8, and high IL-8 expression has been associated with poorer overall survival (25, 26). IL-8 overexpression can also enhance adhesion, migration, invasion, and chemoresistance in gastric cancer cells (27).

We observed concurrent elevation of IL-10 and IL-16, which may have important biological implications. IL-10 is a multifunctional immunoregulatory cytokine that can suppress antigen-presenting cell function, attenuate effector T-cell responses, and enhance regulatory T-cell activity, thereby contributing to an immunosuppressive microenvironment and facilitating tumor immune escape (28, 29). IL-16, primarily produced by CD8+ cells, induces chemotaxis of CD4+ T cells, monocytes, and eosinophils and modulates recruitment and activation of CD4+ cells at inflammatory sites; it was first identified as a chemotactic factor in 1982 (30, 31). Synchronous upregulation of IL-10 and IL-16 may therefore cooperatively promote local immunosuppression in GC, consistent with prior reports linking IL-16 to increased risk of non-cardia gastric cancer and suggesting that the IL-10/IL-16 axis warrants further functional investigation (32). However, the current association analyses were exploratory and based on unadjusted Spearman coefficients. Therefore, these associations should not be interpreted as evidence of direct biological co-regulation independent of shared clinical covariates. Future studies should incorporate covariate-adjusted partial correlation networks and DAG-informed confounder selection to better distinguish direct from confounded relationships.

IL-17 appears to play a biphasic and stage-dependent role during gastric carcinogenesis. In early H. pylori infection, IL-17 contributes to antibacterial defense. Under chronic inflammatory conditions, however, IL-17 can activate NF-κB, STAT3, and related pathways, thereby driving pathological angiogenesis, epithelial–mesenchymal transition, and chemotherapy resistance, linking chronic inflammation to malignant progression (33–36). In the present study, IL-17 remained elevated in both the GPL and GC stages and appeared to shift functionally across disease progression. Nevertheless, our additional analyses suggest that IL-17 should be interpreted in a comparison-specific rather than universal manner. Although IL-17 showed strong discriminatory performance for GC versus HC, the *post-hoc* power analysis indicated only borderline statistical support for GPL versus HC. This pattern, together with its known context- and stage-dependent biology, suggests that IL-17 may not change linearly across all stages of gastric carcinogenesis. Rather, it may reflect a dynamic interplay among chronic inflammation, mucosal injury, and malignant transformation.

Compared with IL-17, IL-10 exhibited a more subtle but still meaningful pattern. Overall effect-size analysis showed a moderate group effect (ϵ² = 0.096), and pairwise Cliff’s delta indicated a medium effect for GC versus HC, a large effect for GPL versus HC, and only a small effect for GC versus GPL. These findings suggest that IL-10 should not be regarded as a null signal. Instead, IL-10 appears to be more effective at distinguishing disease states (GPL or GC) from healthy controls than at distinguishing GC from GPL, indicating that its role may be more closely linked to disease-associated inflammatory or immunoregulatory activation than to fine discrimination between precancerous and malignant stages.

Conversely, several protective or antagonistic immune factors declined during GC progression. IL-7, which is essential for maintaining naïve and memory T-cell homeostasis, was significantly reduced in the GC group, suggesting impaired T-cell surveillance in the tumor microenvironment and supporting an immune-escape phenotype (37). IL-1 receptor antagonist (IL-1ra), the endogenous inhibitor of IL-1 signaling, was also decreased. Such reduction may weaken negative regulation of IL-1-mediated proinflammatory signaling, thereby contributing to unchecked inflammation that favors tumor growth (38, 39). TNF-α, which can regulate angiogenesis-related factors and thereby promote tumor neovascularization, was significantly elevated in GC. However, within the tumor microenvironment, the balance between TNF-α-mediated proapoptotic and prosurvival signaling is often disrupted, with a net shift toward proinflammatory and oncogenic pathways that support disease progression (40, 41).

Serum tumor markers including CEA, CA125, CA19-9, and CA72–4 were significantly elevated in patients with GC but did not differ between individuals with GPL and healthy controls, which is consistent with their limited sensitivity in early-stage disease (8, 42). Nevertheless, CEA retained modest discriminatory value for GC versus GPL (AUC = 0.71), indicating some usefulness for identifying more advanced lesions. A major contribution of the present study lies in the development and validation of multimarker diagnostic models. Individual biomarkers provided useful information, but their diagnostic utility was limited when used alone. IL-16, for example, showed high accuracy for distinguishing GC from HC (AUC = 0.91), suggesting that it may serve as a sensitive serum biomarker across disease progression. Importantly, combining multiple markers substantially improved performance: the IL-6 + IL-7 + IL-16 + CEA model yielded an AUC of 0.93 for GC versus GPL; the IL-6 + IL-16 + IL-17 model achieved an AUC of 0.92 for GPL versus HC; and a 12-analyte composite model showed near-perfect discrimination for GC versus HC (AUC = 0.97). These findings support a combined “inflammatory cytokine profile + tumor marker” strategy as a promising noninvasive approach for early GC screening and risk stratification. However, because highly parameterized models in moderate-sized datasets are vulnerable to overfitting, we performed bootstrap-based internal validation. All combined models retained favorable AUCs after optimism correction, suggesting that their apparent performance was not solely attributable to excessive optimism. Even so, some degree of overfitting risk remains, and these findings should be interpreted cautiously until external validation becomes available.

To improve interpretability of the 12-analyte model, we further performed SHAP-based variable-importance analysis. This showed that IL-16, IL-6, CEA, and IL-7 were the dominant contributors to model output, whereas IL-21 and AFP contributed relatively little. This observation is important because it suggests that the full 12-marker panel may include variables with limited marginal utility. In future studies, a reduced model excluding low-contribution variables may preserve predictive performance while improving clinical practicality. In this context, dimensionality reduction and feature-selection strategies, including penalized regression, correlation pruning, and more advanced machine-learning approaches, should be considered in larger validation cohorts.

The correlation analyses further support this point. After FDR correction, only a limited number of pairwise correlations remained statistically significant, indicating that the correlation structure was substantially sparser than would be inferred from unadjusted heatmap inspection alone. Strong retained pairs, such as IL-16/IL-10 and CEA/CA72-4, may reflect partially overlapping information content. However, correlation alone should not be used as the sole criterion for variable removal, because correlated variables may still provide nonredundant predictive information in multivariable models. Therefore, future panel optimization should combine FDR-adjusted pruning, variable-importance ranking, and predictive validation.

In addition, recent methodological advances suggest that future external validation studies should consider interaction-aware and penalized modeling strategies, such as LASSO (least absolute shrinkage and selection operator), to address high-dimensional biomarker data and reduce overfitting risk (43). Such approaches are particularly valuable when the number of candidate biomarkers is relatively large compared with the sample size, because they simultaneously perform variable selection and regularization, thereby improving model generalizability. More advanced feature-selection frameworks, such as transformer-based weakly supervised learning approaches developed in computational pathology (44), may also be explored in the future to capture complex nonlinear interactions among biomarkers without relying entirely on prespecified biological assumptions. These approaches may further improve the robustness and interpretability of multimarker panels in future multicenter validation studies. Although AUC provides a useful summary measure of discrimination, it does not by itself define the optimal operating threshold for clinical use. In the present study, Youden-optimized cutoffs differed across GC versus GPL, GPL versus HC, and GC versus HC, indicating that threshold selection is comparison-specific rather than universal. In addition, sensitivity at 90% specificity was often substantially lower than the unconstrained optimum implied by the Youden index. This emphasizes the practical tradeoff between false-positive control and case detection and suggests that clinically constrained operating points are essential when considering translation of biomarker panels into screening workflows. To address this issue, future studies should adopt more clinically oriented ROC optimization frameworks, for example by defining thresholds according to acceptable false-positive rates or cost-sensitive decision strategies rather than relying solely on statistically optimized cutoffs (45). Such approaches may better align biomarker-based decision rules with real-world clinical priorities, including resource allocation, patient acceptability, and the consequences of false-negative and false-positive results.

Several limitations of the present study should be emphasized. First, this was a single-center case–control study. Although baseline characteristics were generally balanced across groups, the single-center design limits generalizability and may introduce selection bias. Therefore, the present findings should be regarded as hypothesis-generating and require validation in external multicenter cohorts. Such studies may either confirm the current cytokine progression pattern or refine it by revealing population-specific, molecular-subtype–specific, or infection-context–specific heterogeneity.

Second, the study was cross-sectional. Because no longitudinal follow-up or incident GPL surveillance was included, the observed cytokine differences cannot establish temporal causality. It remains unclear whether these biomarker shifts precede lesion development, accompany disease progression, or simply reflect established disease at the time of sampling. In addition, because prevalent rather than incident cases were enrolled, prevalence-incidence bias cannot be excluded. Longitudinal studies with repeated cytokine measurements across the transition from GPL to GC are needed to define the temporal trajectory and causal relevance of these biomarkers.

Third, although BMI categories and H. pylori infection status were balanced across groups, residual confounding cannot be fully excluded. BMI was used descriptively rather than as a continuous covariate, and H. pylori status was not incorporated into the current cytokine–tumor marker modeling framework. This issue may be particularly relevant for cytokines such as IL-6 and IL-8, which are known to be inducible by H. pylori. Future studies should incorporate continuous BMI, body-composition measures, nutritional indices, and H. pylori-adjusted or stratified analyses to better distinguish disease-related effects from infection- or host-related inflammatory effects.

Fourth, although AG and IM occupy distinct positions along the Correa cascade, exploratory subgroup analyses in the present cohort did not reveal statistically significant differences in cytokines or tumor markers between these two GPL subgroups. This provides some support for the pooled GPL analysis used in the main study. However, this should not be interpreted as evidence of biological equivalence, because subtle stage-dependent differences may still exist and may require larger stage-resolved cohorts to detect, particularly for cytokines such as IL-17. Similarly, exploratory comparison of HGIN/carcinoma *in situ* and invasive GC did not reveal statistically significant serum biomarker differences in the current dataset. This supports the practicality of pooled GC analysis in the current study, but not biological equivalence between these disease states. Finally, although tumor marker values were obtained from routine clinical records rather than prospectively remeasured within the study protocol, all values were derived from the same institutional laboratory system, which minimized cross-platform technical heterogeneity. Nevertheless, assay harmonization remains an important consideration for future multicenter validation studies.

Overall, the present study identifies several cytokines and multimarker panels with promising discriminatory value across GC, GPL, and HC. However, these findings should be interpreted as exploratory and should be validated in larger multicenter, longitudinal, and stage-resolved cohorts before being considered for clinical implementation.

## 5 Conclusion

This study identified several cytokines and multimarker panels with promising discriminatory value across GC, GPL, and HC. In particular, IL-16, IL-6, IL-7, and IL-17 emerged as informative biomarkers, and their integration with conventional tumor markers further improved diagnostic performance. However, these findings should be interpreted as exploratory. Larger multicenter, longitudinal, and stage-resolved studies are required to validate these biomarkers, refine model structure, and determine their practical value in early screening and risk stratification.

## Data Availability

The raw data supporting the conclusions of this article will be made available by the authors, without undue reservation.

## References

[1] Bray F Laversanne M Sung H Ferlay J RL S Soerjomataram I . Global cancer statistics 2022: GLOBOCAN estimates of incidence and mortality worldwide for 36 cancers in 185 countries. CA Cancer J Clin. (2024) 74:229–63. doi: 10.3322/caac.21834, PMID: 38572751

[2] Health Commission Of The People's Republic Of China N . National guidelines for diagnosis and treatment of gastric cancer 2022 in China (English version). Chin J Cancer Res. (2022) 34:207–37. 10.21147/j.issn.1000-9604.2022.03.04 PMC9273576 35873885

[3] Ibrahim A Morais S Ferro A Lunet N Peleteiro B . Sex-differences in the prevalence of Helicobacter pylori infection in pediatric and adult populations: Systematic review and meta-analysis of 244 studies. Dig. Liver Dis. (2017) 49:742–9. doi: 10.1016/j.dld.2017.03.019, PMID: 28495503

[4] Ravla P Barsouk A . Epidemiology of gastric cancer: Global trends, risk factors and prevention. Prz. Gastroenterol. (2019) 14:26–38. doi: 10.5114/pg.2018.80001, PMID: 30944675 PMC6444111

[5] Wesołowska M Pawlik P Jagodziński PP . The clinicopathologic significance of estrogen receptors in human gastric carcinoma. BioMed Pharmacother. (2016) 83:314–22. doi: 10.1016/j.biopha.2016.06.048, PMID: 27399808

[6] Correa P Haenszel W Cuello C Tannenbaum S Archer M . A model for gastric cancer. Lancet. (1975) 2:58–60. 49653 10.1016/s0140-6736(75)90498-5

[7] Akbari M Tabrizi R Kardeh S Lankarani KB . Gastric cancer in patients with gastric atrophy and intestinal metaplasia: A systematic review and meta-analysis. PloS One. (2019) 14:e0219865. doi: 10.1371/journal.pone.0219865, PMID: 31348819 PMC6660080

[8] Shimada H Noie T Ohashi M Oba K Takahashi Y . Clinical significance of serum tumor markers for gastric cancer: a systematic review of literature by the Task Force of the Japanese Gastric Cancer Association. Gastric Cancer. (2014) 17:26–33. doi: 10.1007/s10120-013-0259-5, PMID: 23572188

[9] Khandia R Munjal A . Interplay between inflammation and cancer. Adv Protein Chem Struct Biol. (2020) 119:199–245. doi: 10.1016/bs.apcsb.2019.09.004, PMID: 31997769

[10] Elinav E Nowarski R Thaiss CA Hu B Jin C Flavell RA . Inflammation-induced cancer: crosstalk between tumours, immune cells and microorganisms. Nat Rev Cancer. (2013) 13:759–71. doi: 10.1038/nrc3611, PMID: 24154716

[11] Propper DJ Balkwill FR . Harnessing cytokines and chemokines for cancer therapy. Nat Rev Clin Oncol. (2022) 19:237–53. doi: 10.1038/s41571-021-00588-9, PMID: 34997230

[12] Lan T Chen L Wei X . Inflammatory cytokines in cancer: comprehensive understanding and clinical progress in gene therapy. Cells. (2021) 10:100. doi: 10.3390/cells10010100, PMID: 33429846 PMC7827947

[13] Ock CY Nam AR Bang JH Kim TY Lee KH Han SW . Signature of cytokines and angiogenic factors (CAFs) defines a clinically distinct subgroup of gastric cancer. Gastric Cancer. (2017) 20:164–74. doi: 10.1007/s10120-015-0583-z, PMID: 26681196

[14] Macrì A Versaci A Loddo S Scuderi G Travagliante M Trimarchi G . Serum levels of interleukin 1beta, interleukin 8 and tumour necrosis factor alpha as markers of gastric cancer. Biomarkers. (2006) 11:184–93. doi: 10.1080/13547500600565677, PMID: 16766394

[15] Yarmohammadi R Najafi K Noroozbeygi M Didehvar K Rastin A Ataei F . The role of IL-6, IL-10 and CRP in gastrointestinal cancers. Cell Biol Int. (2025) 49:1061–78. doi: 10.1002/cbin.70050, PMID: 40590606

[16] Richmond J Tuzova M Cruikshank W Center D . Regulation of cellular processes by interleukin-16 in homeostasis and cancer. J Cell Physiol. (2014) 229:139–47. doi: 10.1002/jcp.24441, PMID: 23893766

[17] Koh CH Kim BS Kang CY Chung Y Seo H . IL-17 and IL-21: their immunobiology and therapeutic potentials. Immune Netw. (2024) 24:e2. doi: 10.4110/in.2024.24.e2, PMID: 38455465 PMC10917578

[18] Huang B Lang X Li X . The role of IL-6/JAK2/STAT3 signaling pathway in cancers. Front Oncol. (2022) 12:1023177. doi: 10.3389/fonc.2022.1023177, PMID: 36591515 PMC9800921

[19] Ham IH Oh HJ Jin H Bae CA Jeon SM Choi KS . Targeting interleukin-6 as a strategy to overcome stroma-induced resistance to chemotherapy in gastric cancer. Mol Cancer. (2019) 18:68. doi: 10.1186/s12943-019-0972-8, PMID: 30927911 PMC6441211

[20] Abaurrea A Araujo AM Caffarel MM . The role of the IL-6 cytokine family in epithelial-mesenchymal plasticity in cancer progression. Int J Mol Sci. (2021) 22:8334. doi: 10.3390/ijms22158334, PMID: 34361105 PMC8347315

[21] Yu B Xiang L Peppelenbosch MP Fuhler GM . Overlapping cytokines in H. pylori infection and gastric cancer: A tandem meta-analysis. Front Immunol. (2023) 14:1125658. doi: 10.3389/fimmu.2023.1125658, PMID: 37006300 PMC10050690

[22] Yu B de Vos D Guo X Peng S Xie W Peppelenbosch MP . IL-6 facilitates cross-talk between epithelial cells and tumor- associated macrophages in Helicobacter pylori-linked gastric carcinogenesis. Neoplasia. (2024) 50:100981. doi: 10.1016/j.neo.2024.100981, PMID: 38422751 PMC10912637

[23] Fousek K Horn LA Palena C . Interleukin-8: A chemokine at the intersection of cancer plasticity, angiogenesis, and immune suppression. Pharmacol Ther. (2021) 219:107692. doi: 10.1016/j.pharmthera.2020.107692, PMID: 32980444 PMC8344087

[24] Macedo F Ladeira K Longatto-Filho A Martins SF . Gastric cancer and angiogenesis: is VEGF a useful biomarker to assess progression and remission? J Gastric Cancer. (2017) 17:1–10. doi: 10.5230/jgc.2017.17.e1, PMID: 28337358 PMC5362829

[25] Li W Lin S Li W Wang W Li X Xu D . IL-8 interacts with metadherin promoting proliferation and migration in gastric cancer. Biochem Biophys Res Commun. (2016) 478:1330–7. doi: 10.1016/j.bbrc.2016.08.123, PMID: 27565732

[26] Lee KE Khoi PN Xia Y Park JS Joo YE Kim KK . Helicobacter pylori and interleukin-8 in gastric cancer. World J Gastroenterol. (2013) 19:8192–202. doi: 10.3748/wjg.v19.i45.8192, PMID: 24363509 PMC3857441

[27] Kuai WX Wang Q Yang XZ Zhao Y Yu R Tang XJ . Interleukin-8 associates with adhesion, migration, invasion and chemosensitivity of human gastric cancer cells. World J Gastroenterol. (2012) 18:979–85. doi: 10.3748/wjg.v18.i9.979, PMID: 22408359 PMC3297059

[28] Ouyang W O'Garra A . IL-10 family cytokines IL-10 and IL-22: from basic science to clinical translation. Immunity. (2019) 50:871–91. doi: 10.1016/j.immuni.2019.03.020, PMID: 30995504

[29] Carlini V Noonan DM Abdalalem E Goletti D Sansone C Calabrone L . The multifaceted nature of IL-10: regulation, role in immunological homeostasis and its relevance to cancer, COVID-19 and post-COVID conditions. Front Immunol. (2023) 14:1161067. doi: 10.3389/fimmu.2023.1161067, PMID: 37359549 PMC10287165

[30] Center DM Cruikshank W Identification I . and characterization of chemoattractant activity for lymphocytes from mitogen-stimulated mononuclear cells. J Immunol. (1982) 128:2563–8. 7042840

[31] Alexandrakis MG Passam FH Kyriakou DS Christophoridou AV Perisinakis K Hatzivasili A . Serum level of interleukin-16 in multiple myeloma patients and its relationship to disease activity. Am J Hematol. (2004) 75:101–6. doi: 10.1002/ajh.10444, PMID: 14755377

[32] Zhang T Wang H . Variants of interleukin-16 associated with gastric cancer risk. Asian Pac J Cancer Prev. (2013) 14:5269–73. doi: 10.7314/apjcp.2013.14.9.5269, PMID: 24175812

[33] Al-Shawk RS Bakir WAE Mohammed ZA . Exploring IL-17 and IL-23 as biomarkers in H. pylori-linked gastric diseases: A cross-sectional study. Microb Pathog. (2025) 206:107741. doi: 10.1016/j.micpath.2025.107741, PMID: 40441389

[34] Brackman LC Jung MS Green EH Joshi N Revetta FL McClain MS . IL-17 signaling protects against Helicobacter pylori-induced gastric cancer. Gut Microbes. (2024) 16:2430421. doi: 10.1080/19490976.2024.2430421, PMID: 39588838 PMC11639209

[35] Miossec P Kolls JK . Targeting IL-17 and TH17 cells in chronic inflammation. Nat Rev Drug Discov. (2012) 11:763–76. doi: 10.1038/nrd3794, PMID: 23023676

[36] Cua DJ Tato CM . Innate IL-17-producing cells: the sentinels of the immune system. Nat Rev Immunol. (2010) 10:479–89. doi: 10.1038/nri2800, PMID: 20559326

[37] Mazzucchelli R Durum SK . Interleukin-7 receptor expression: intelligent design. Nat Rev Immunol. (2007) 7:144–54. doi: 10.1038/nri2023, PMID: 17259970

[38] Xiyue X Dan L Ming S . Research progress on the relationship between IL-1β and tumor Malignant progression. Chin J Immunol. (2020) 36:1387–91.

[39] Liu J Wei F Zhao W Lu N Liu X . Protective effect of IL-1Ra on airway inflammation and airway remodeling in COPD model rats. China Med Herald. (2019) 16:21–5.

[40] Hammam O Mahmoud O Zahran M Sayed A Salama R Hosny K . A possible role for TNF-α in coordinating inflammation and angiogenesis in chronic liver disease and hepatocellular carcinoma. Gastrointest Cancer Res. (2013) 6:107–14. PMC3782877 24147158

[41] Balkwill F . Tumour necrosis factor and cancer. Nat Rev Cancer. (2009) 9:361–71. doi: 10.1038/nrc2628, PMID: 19343034

[42] Liu X Qiu H Liu J Chen S Xu D Li W . Combined preoperative concentrations of CEA, CA 19-9, and 72–4 for predicting outcomes in patients with gastric cancer after curative resection. Oncotarget. (2016) 7:35446–53. doi: 10.18632/oncotarget.9060, PMID: 27147574 PMC5085242

[43] Liu B Li C He S Li Z Wang H Feng C . Ubiquitin-conjugating enzyme E2S (UBE2S) as a prognostic biomarker and regulator of tumorigenesis in osteosarcoma. Int Immunopharmacol. (2025) 154:114545. doi: 10.1016/j.intimp.2025.114545, PMID: 40188527

[44] Jiang R Yin X Yang P Cheng L Hu J Yang J . A transformer-based weakly supervised computational pathology method forclinical-grade diagnosis and molecular marker discovery of gliomas. Nat Mach Intell. (2024) 6:876–91. doi: 10.1038/s42256-024-00868-w, PMID: 37880705

[45] Peng W Chen L Liu J . Celastrol inhibits gastric cancer cell proliferation, migration, and invasion via the FOXA1/CLDN4 axis. Toxicol Res (Camb). (2023) 12:392–9. 10.1093/toxres/tfad024 PMC10311132 37397926

