# Supplementary material for: Development of an early diagnostic model for gastric cancer combining serum cytokine profiles with conventional tumor markers: a case–control study
Source: Front Oncol. 2026 Apr 28;16:1804938. doi: 10.3389/fonc.2026.1804938 (PMC13160785; doi:10.3389/fonc.2026.1804938)
Supplement: Supplementary file 1 [file DataSheet1.docx]

**Supplementary materials**

**Supplementary Table S1**. Quality-control metrics for ELISA-based quantification of the nine serum cytokines

| Biomarker | samples tested | Duplicate assay CV (%) | Re-assay rate (%) | Recovery range (%) | Mean recovery (%) | Sample handling condition | QC conclusion |
| --- | --- | --- | --- | --- | --- | --- | --- |
| IL-1ra | 165 | 7.8 | 3.0 | 88.4-108.7 | 97.2 | Stored at −80°C; analyzed in one session | Acceptable |
| IL-6 | 165 | 6.9 | 2.4 | 90.1-109.5 | 98.4 | Stored at −80°C; analyzed in one session | Acceptable |
| IL-7 | 165 | 8.5 | 3.6 | 86.7-111.2 | 96.8 | Stored at −80°C; analyzed in one session | Acceptable |
| IL-8 | 165 | 7.1 | 2.4 | 89.6-107.4 | 97.9 | Stored at −80°C; analyzed in one session | Acceptable |
| IL-10 | 165 | 9.2 | 4.2 | 85.9-110.3 | 96.1 | Stored at −80°C; analyzed in one session | Acceptable |
| IL-16 | 165 | 6.5 | 1.8 | 91.3-106.8 | 99.1 | Stored at −80°C; analyzed in one session | Acceptable |
| IL-17 | 165 | 8.9 | 3.6 | 87.1-109.8 | 97.0 | Stored at −80°C; analyzed in one session | Acceptable |
| IL-21 | 165 | 8.1 | 3.0 | 88.0-108.2 | 96.9 | Stored at −80°C; analyzed in one session | Acceptable |
| TNF-α | 165 | 7.4 | 2.4 | 89.2-107.9 | 98.0 | Stored at −80°C; analyzed in one session | Acceptable |

Footnote:Values shown are summary quality-control metrics for each cytokine assay. Duplicate assay CV was calculated from paired measurements. Samples exceeding the predefined acceptance threshold were re-assayed. Spike-in recovery was evaluated in representative serum samples. All serum samples were processed according to a standardized workflow, stored at −80°C, and analyzed together under uniform experimental conditions.

**Supplementary Table S2**. Post-hoc power assessment for ROC analyses of selected biomarkers

| Biomarker | Comparison | n (case/control) | AUC (95% CI) | Estimated power for AUC > 0.70 (%) | Interpretation |
| --- | --- | --- | --- | --- | --- |
| IL-6 | GC vs GPL | 60/50 | 0.74(0.66-0.84) | 81 | Adequate |
| IL-7 | GC vs GPL | 60/50 | 0.76(0.67-0.85) | 90 | Adequate |
| IL-16 | GC vs GPL | 60/50 | 0.75(0.64-0.82) | 86 | Adequate |
| CEA | GC vs GPL | 60/50 | 0.71(0.62-0.81) | 58 | Limited |
| IL-6 | GPL vs HC | 50/55 | 0.72(0.66-0.84) | 68 | Borderline |
| IL-16 | GPL vs HC | 50/55 | 0.74(0.62-0.82) | 77 | Borderline |
| IL-17 | GPL vs HC | 50/55 | 0.73(0.64-0.82) | 74 | Borderline |
| IL-6 | GC vs HC | 60/55 | 0.80(0.72-0.88) | 99 | Strong |
| IL-8 | GC vs HC | 60/55 | 0.74(0.65-0.83) | 81 | Adequate |
| IL-7 | GC vs HC | 60/55 | 0.81(0.73-0.89) | 99 | Strong |
| IL-10 | GC vs HC | 60/55 | 0.72(0.63-0.82) | 66 | Borderline |
| IL-16 | GC vs HC | 60/55 | 0.91(0.86-0.96) | >99 | Strong |
| IL-17 | GC vs HC | 60/55 | 0.83(0.76-0.91) | >99 | Strong |
| IL-21 | GC vs HC | 60/55 | 0.70(0.64-0.82) | 50 | Limited |
| IL-1ra | GC vs HC | 60/55 | 0.72(0.61-0.80) | 66 | Borderline |
| TNF-α | GC vs HC | 60/55 | 0.75(0.66-0.84) | 86 | Adequate |
| CA125 | GC vs HC | 60/55 | 0.72(0.62-0.81) | 66 | Borderline |
| CA199 | GC vs HC | 60/55 | 0.71(0.60-0.82) | 57 | Limited |
| CEA | GC vs HC | 60/55 | 0.80(0.72-0.88) | >99 | Strong |

Footnote:Post-hoc power was estimated from the observed AUC values and their corresponding 95% confidence intervals for each biomarker-comparison pair. Power estimates refer to testing whether the AUC exceeded 0.70 at a one-sided alpha level of 0.05. These analyses were performed to support interpretation of statistical precision and do not replace a prespecified a priori sample size calculation. The labels “Strong,” “Adequate,” “Borderline,” and “Limited” are descriptive only.

**Supplementary Table S3** Overall effect sizes for Kruskal–Wallis tests across GC, GPL, and HC

| Biomarker | Kruskal–Wallis H (K) | P Value | Epsilon-squared (ε²) | Magnitude |
| --- | --- | --- | --- | --- |
| CA125 | 18.521 | 0.000095 | 0.102 | Moderate |
| CA199 | 17.663 | 0.000146 | 0.096 | Moderate |
| CA724 | 6.683 | 0.035 | 0.029 | Small |
| CEA | 36.917 | 9.6304E-9 | 0.216 | Moderate-to-large |
| AFP | 1.801 | 0.406 | <0.001 | Negligible |

Continued Supplementary Table S3 Overall effect sizes for Kruskal–Wallis tests across GC, GPL, and HC

| IL-16 | 61.457 | 4.5171E-14 | 0.367 | Large |
| --- | --- | --- | --- | --- |
| IL-6 | 36.756 | 1.0434E-8 | 0.214 | Moderate-to-large |
| IL-8 | 23.428 | 0.000008 | 0.132 | Moderate |
| IL-10 | 17.517 | 0.000157 | 0.096 | Moderate-to-large |
| IL-17 | 34.136 | 3.8675E-8 | 0.198 | Moderate-to-large |
| TNF-α | 21.400 | 0.000023 | 0.120 | Moderate |
| IL-7 | 41.387 | 1.0301E-9 | 0.244 | Moderate-to-large |
| IL-21 | 18.302 | 0.000106 | 0.101 | Moderate |
| IL-1ra | 15.737 | 0.000383 | 0.085 | Moderate |

Footnote:Epsilon-squared (ε²) was calculated as the overall effect-size estimate for Kruskal–Wallis tests using the formula ε² = (H − k + 1)/(N − k), where H is the Kruskal–Wallis statistic, k is the number of groups, and N is the total sample size. Effect sizes were interpreted using conventional thresholds as small (~0.01), moderate (~0.08), and large (~0.26).

**Supplementary Table S4** Pairwise Cliff’s delta for IL-10 across GC, GPL, and HC

| Biomarker | Comparison | n | Cliff’s delta | Magnitude | Direction |
| --- | --- | --- | --- | --- | --- |
| IL-10 | GC vs HC | 60/55 | 0.383 | Medium | Higher in GC |
| IL-10 | GPL vs HC | 50/55 | 0.596 | Large | Higher in GPL |
| IL-10 | GC vs GPL | 60/50 | -0.242 | Small | Higher in GPL |

Footnote:Cliff’s delta was used to quantify pairwise effect sizes for non-normally distributed IL-10 levels. Positive values indicate higher values in the first-listed group, whereas negative values indicate higher values in the second-listed group. Magnitudes were interpreted using conventional thresholds: negligible (<0.147), small (0.147–0.330), medium (0.330–0.474), and large (≥0.474).

**Supplementary Table S5** Comparison of serum cytokine levels between atrophic gastritis (AG) and intestinal metaplasia (IM)

| Biomarker | AG (n=25) Median (IQR) | IM (n=25) Median (IQR) | Raw P-value | Adjusted q-value |
| --- | --- | --- | --- | --- |
| IL-6 | 2.58 (1.45–4.67) | 3.70 (2.30–6.70) | 0.123 | 0.287 |
| IL-7 | 5.00 (4.20–6.50) | 4.64 (4.17–5.70) | 0.312 | 0.473 |
| IL-8 | 15.69 (8.89–21.70) | 14.60 (9.40–19.77) | 0.672 | 0.783 |
| IL-10 | 12.54 (9.67–19.60) | 11.98 (8.43–14.60) | 0.241 | 0.375 |
| IL-16 | 12.56 (8.63–22.50) | 10.97 (7.43–17.23) | 0.371 | 0.495 |
| IL-17 | 21.82 (17.40–36.35) | 21.98 (12.85–33.60) | 0.784 | 0.891 |
| IL-21 | 55.78 (48.50–67.70) | 55.40 (48.40–64.67) | 0.823 | 0.881 |
| IL-1ra | 216.4 (146.7–324.6) | 245.6 (173.6–320.6) | 0.435 | 0.554 |
| TNF-α | 14.17 (11.36–23.17) | 15.20 (11.09–22.30) | 0.654 | 0.783 |

Footnote:Data are presented as median (interquartile range). P-values were calculated using the Mann–Whitney U test. Adjusted q-values were obtained using the Benjamini–Hochberg false discovery rate (FDR) correction for multiple comparisons. All cytokine concentrations are in pg/mL.

**Supplementary Table S6** Comparison of serum tumor marker levels between AG and IM groups.

| Biomarker | AG (n=25) Median (IQR) | IM (n=25) Median (IQR) | Raw P-value | Adjusted q-value |
| --- | --- | --- | --- | --- |
| CA125 | 10.6 (7.0–15.2) | 8.9 (6.4–12.2) | 0.289 | 0.437 |
| CA199 | 9.6 (5.5–19.2) | 8.3 (5.0–12.8) | 0.411 | 0.554 |
| CA724 | 4.6 (1.5–8.5) | 4.4 (1.3–7.6) | 0.638 | 0.783 |
| CEA | 2.6 (1.1–4.8) | 1.6 (1.0–2.7) | 0.092 | 0.257 |
| AFP | 3.2 (2.0–5.4) | 2.9 (1.6–4.6) | 0.487 | 0.605 |

Footnote:Data are presented as median (interquartile range). P-values were calculated using the Mann–Whitney U test. Adjusted q-values were obtained using the Benjamini–Hochberg FDR correction. Tumor marker concentrations are in U/mL for CA125, CA199, CA724, and in ng/mL for CEA and AFP.

**Supplementary Table S7** Bootstrap internal validation results of the combined biomarker models

| Comparison | Model composition | Apparent AUC | Average optimism | Optimism-corrected | AUC 95% CI |
| --- | --- | --- | --- | --- | --- |
| GC vs GPL | IL-6+IL-7+IL-16+CEA | 0.93 | 0.028 | 0.902 | 0.857-0.941 |
| GPL vs HC | IL-6+IL-16+IL-17 | 0.92 | 0.031 | 0.889 | 0.834-0.927 |
| GC vs HC | IL-6+IL-8+IL-7+IL-10+IL-16+IL-17+IL-21+IL-1ra+TNF-α+CA125+CA199+CEA | 0.97 | 0.043 | 0.927 | 0.891-0.965 |

Footnote:Bootstrap resampling was performed 1,000 times. Apparent AUC values were derived from the original combined biomarker models shown in Table 7. Optimism-corrected AUC was calculated as the apparent AUC minus the average optimism. The 95% confidence intervals were obtained using the percentile method.

**Supplementary Table S8** SHAP-based variable importance for the 12-analyte model discriminating gastric cancer from healthy controls

| Rank | Biomarker | Mean SHAP Value(95% CI) | Relative Importance (%) |
| --- | --- | --- | --- |
| 1 | IL-16 | 0.85 (0.72-0.98) | 21.3% |
| 2 | IL-6 | 0.72 (0.61-0.83) | 18.0% |
| 3 | CEA | 0.68 (0.55-0.81) | 17.0% |
| 4 | IL-7 | 0.59 (0.48-0.70) | 14.8% |
| 5 | IL-17 | 0.35 (0.26-0.44) | 8.8% |
| 6 | TNF-α | 0.22 (0.15-0.29） | 5.5% |
| 7 | IL-8 | 0.18 (0.11-0.25） | 4.5% |
| 8 | CA125 | 0.15 (0.09-0.21） | 3.8% |
| 9 | IL-10 | 0.12 (0.06-0.18） | 3.0% |
| 10 | CA199 | 0.08 (0.03-0.13） | 2.0% |
| 11 | IL-21 | 0.05 (0.01-0.09） | 1.3% |
| 12 | AFP | 0.02 (-0.01-0.05） | 0.5% |
| 13 | Total | 4.00 | 100% |

Footnote:SHAP (Shapley additive explanations) values were used to quantify the relative contribution of each biomarker to the output of the 12-analyte classification model for GC versus HC. Higher mean SHAP values indicate greater contribution to model prediction.

**Supplementary Table S9** Comparison of serum biomarker levels between patients with high-grade intraepithelial neoplasia (HGIN)/carcinoma in situ and invasive gastric cancer

| Biomarker | HGIN (n=15) Median (IQR) | Invasive GC (n=45) Median (IQR) | P-value | Adjusted P-value |
| --- | --- | --- | --- | --- |
| IL-6 | 11.2 (8.5-15.8) | 13.1 (9.2-18.7) | 0.23 | 0.68 |
| IL-7 | 3.8 (3.1-4.5) | 3.6 (2.9-4.8) | 0.81 | 0.94 |
| IL-8 | 28.4 (16.8-41.2) | 32.1 (18.5-45.6) | 0.34 | 0.72 |
| IL-10 | 15.2 (11.3-20.5) | 17.8 (12.9-24.1) | 0.19 | 0.65 |
| IL-16 | 42.5 (30.1-58.7) | 48.9 (35.6-65.4) | 0.15 | 0.61 |
| IL-17 | 48.3(35.6-66.7) | 55.4(40.2-74.5) | 0.21 | 0.66 |
| Il-21 | 109.5 (95.2-128.7) | 117.8(99.5-138.2) | 0.28 | 0.70 |
| IL-1ra | 225.6 (170.3-310.5) | 270.4 (185.7-360.8) | 0.31 | 0.71 |
| TNF-α | 18.4 (12.5-25.6) | 21.5 (14.7-30.1) | 0.17 | 0.63 |
| CEA | 2.9(1.5-5.8) | 3.5(1.8-8.2) | 0.42 | 0.78 |
| CA125 | 19.8 (12.1-35.4) | 24.5(14.6-41.2) | 0.26 | 0.69 |
| CA199 | 18.5 (8.9-42.7) | 23.6 (11.2-55.8) | 0.33 | 0.72 |
| CA724 | 4.8 (1.9-12.5) | 5.9(2.2-16.8) | 0.48 | 0.82 |
| AFP | 2.9(1.8-4.2) | 3.1(1.9-4.8) | 0.62 | 0.89 |

Footnote:P values were calculated using the Mann–Whitney U test. Adjusted P values were obtained using Bonferroni correction for multiple comparisons. Abbreviations: IQR, interquartile range; GC, gastric cancer; HGIN, high-grade intraepithelial neoplasia.
